# Diazotroph Diversity Associated With Scleractinian Corals and Its Relationships With Environmental Variables in the South China Sea

**DOI:** 10.3389/fphys.2020.00615

**Published:** 2020-06-17

**Authors:** Jiayuan Liang, Kefu Yu, Yinghui Wang, Xueyong Huang, Wen Huang, Zhenjun Qin, Guanghua Wang, Hongfei Su, Biao Chen, Zhengchao Wu

**Affiliations:** ^1^Coral Reef Research Center of China, Guangxi University, Nanning, China; ^2^Guangxi Laboratory on the Study of Coral Reefs in the South China Sea, Nanning, China; ^3^School of Marine Sciences, Guangxi University, Nanning, China; ^4^State Key Laboratory of Tropical Oceanography (LTO), South China Sea Institute of Oceanology, Chinese Academy of Sciences, Guangzhou, China

**Keywords:** coral reef ecosystem, nitrogen fixation, *nifH* gene, geographical differences, different latitudes

## Abstract

Coral reef ecosystems cannot operate normally without an effective nitrogen cycle. For oligotrophic coral reef areas, coral-associated diazotrophs are indispensable participants in the nitrogen cycle. However, the distribution of these diazotrophs and the correlation with the physical and chemical variables of the surrounding seawater remain unclear. To this end, 68 scleractinian coral colonies were sampled from 6 coral reef areas with different environmental variables in the South China Sea to investigate the composition of associated diazotrophs based on *nifH* gene amplification using high-throughput sequencing. The six coral reefs can be clearly divided into two types (fringing reefs and island reefs), are affected by varying degrees of human activities and are located at different latitudes from 9°20’06”N to 22°34’55”N with different seawater temperatures. Alpha- and beta-diversity analyses showed that the distribution of diazotrophs among coral reefs exhibited significant geographical fluctuations (*p* ≤ 0.05) and non-significant interspecific fluctuations (*p* > 0.05). The predominant bacterial phyla included Proteobacteria, Chlorobi, Cyanobacteria, and two unclassified phyla. Chlorobi exhibited a relative abundance of 47–96% in coral samples from the high-latitude Daya Bay fringing reef affected by eutrophication. Unclassified bacteria II, with a relative abundance of 28–87%, was found in all coral samples from the midlatitude Luhuitou fringing reef affected by eutrophication. However, unclassified bacteria I and Proteobacteria dominated (>80% relative abundance) in most of the coral samples from the Weizhou Island fringing reef, which is far from land, and three island reefs (Huangyan Island, Xinyi Reef, and Sanjiao Reef) at relatively low latitudes. At the genus level, some core diazotrophs were found in different coral sample groups. In addition, correlation analysis with various environmental variables revealed that the variables were positively or negatively correlated with different diazotrophic genera. Coral-associated diazotrophs were common among coral individuals. However, their composition was closely related to the different environmental variables. These results provide insights into the geographical distribution characteristics of coral-associated diazotrophs and their evolutionary trends in response to environmental change in the South China Sea.

## Introduction

Although coral reef ecosystems are located in oligotrophic seas, their biodiversity and primary productivity are extremely high (Connell, 1978). This phenomenon is mainly due to the efficient biogeochemical cycles of carbon, nitrogen, phosphorus, and other basic elements in which coral symbiotic microbes participate (Nakajima et al., 2013). In an ocean environment with very low concentrations of nutrients, the primary productivity of coral reefs is often limited by available nitrogen, which is one of the primary nutrients essential for the survival of all living organisms (Muscatine et al., 1989; Rädecker et al., 2015). The nitrogen fixation system of coral-associated diazotrophs (reduction of N_2_ to ammonia) is considered the major source of available nitrogen in coral reef waters (Piniak et al., 2003; Olson et al., 2009). Previous studies have shown that corals have their own internal nitrogen circulation system and protection mechanism (Crossland and Barnes, 1976; Davey et al., 2008). In addition, abundant nitrogen-fixing bacteria associated with corals have been detected in different coral compartments, including mucus (Wegley et al., 2007; Chimetto et al., 2008), tissue (Lesser et al., 2004; Lema et al., 2012), and skeleton (Crossland and Barnes, 1976; Yang et al., 2016). In addition, some studies predicted that diazotrophs associated with corals not only provided sufficient nitrogen sources for coral holobionts, including coral hosts and all microbial organisms that live with them, but also supplied ~6% of the organic nitrogen for the whole coral reef ecosystem when the available nitrogen was low (Wilkinson et al., 1984; Williams et al., 1987). Recent studies found that coral-associated diazotrophs could significantly respond to human-induced environmental changes, thermal stress, and coral bleaching. For example, the key physiological traits (severe loss of zooxanthellae, net photosynthesis, and N_2_ fixation rates) of coral hosts, zooxanthellae, and diazotrophs associated with *Stylophora pistillata* were found to show less resilience to thermal stress than those associated with *Acropora hemprichii*. In addition, it was found that the N_2_ fixation rate drastically increased in daylight due to high temperature stress, but increased only in the dark after the temperature was reduced again to *in situ* levels. Concurrently, coral hosts, particularly bleached individuals, were found to exhibit reduced organic matter release and heterotrophic feeding on picoplankton. These results indicated that coral-associated diazotrophs play an important role in the responses of the coral holobiont to ocean warming (Cardini et al., 2016). The relationship between the bleaching mortality and nitrogen fixation rates of diazotrophs in the coral *Acropora aspera* showed that the N_2_ fixation rates on coral skeletons following bleaching mortality (caused by thermal or cold bleaching) were up to 30 times greater than those measured in live colonies (Davey et al., 2008).

Currently, the following three main routes are involved in the provision of nitrogen to coral reef ecosystems: (1) terrestrial input, (2) input from ocean currents with rich nutrients, and (3) nitrogen fixation by diazotrophs in coral reef ecosystems (Crossland and Barnes, 1976; Rougerie and Wauthy, 1993; Yamamuro et al., 1995; Sammarco et al., 1999; Sarhan et al., 2000). However, isotope (δ13C and δ15N) tracer experiments have shown that the sources of nitrogen for the primary producers in coral reefs (in Palau and Ishigaki) were mainly derived from biological nitrogen fixation (Yamamuro et al., 1995). Furthermore, the photosynthetic fixation of CO_2_ was found to occur simultaneously with the absorption and fixation of new nitrogen. This finding suggests that biological nitrogen fixation plays an important role in the assimilation of carbon and nitrogen by the whole coral reef ecosystem.

Recently, the diversity of the diazotrophic communities associated with different coral species, including *S. pistillata* and *A. hemprichii* (Cardini et al., 2016), *Cladopsammia gracilis* and *Porites* sp. (Grover et al., 2014), *Montipora capitata* and *Montipora flabellata* (Olson et al., 2009), and others (Lema et al., 2012, 2016; Grover et al., 2014), were investigated. These coral species were investigated in different geographical locations, including the Great Barrier Reef (Kelso Reef, Knife Reef, and Davies Reef), the Luhuitou fringing reef of Sanya Bay (South China Sea), the Marine Science Station in Aqaba (Jordan), the Gulf of Aqaba (Red Sea), Leleiwi Reef (Hawaii Island), and Green Island (southeastern Taiwan). However, the geographical distribution and host specificity of coral-associated diazotrophs as well as the correlation with the physical and chemical variables of the surrounding seawater remain unclear.

In the South China Sea, there is a large area of coral reefs at a latitudinal range of 4–21°N (Yu K. -F. et al., 2004). These coral reefs have long been affected by extreme marine events (El Niño, strong storms, high-frequency winter cooling, etc.), human activities, and varying geographical climates (Yu K. -F. et al., 2004; Yu K. et al., 2004; Yu et al., 2009; Yu, 2012; Zhang et al., 2019). Coral skeletons, as carriers of high-resolution environmental records, clearly recorded the climate mutation events during the Holocene, the intensity of El Niño activity at the millennium scale, sea level fluctuations at the millennium-hundred-year scale, periodic strong wind storm activities, East Asian monsoon records, and information on seawater acidity, pollution status, etc. That is, the existing living corals (including members of the internal symbiotic microorganisms) in the South China Sea have evolved slowly through different environmental stresses. Therefore, coral reefs in the South China Sea are natural laboratories that can be used to study the ecological characteristics of coral-associated diazotrophs. To this end, 68 colonies representing 11 species were collected from 6 geographical locations at different latitudes and with different eutrophication levels in the South China Sea to investigate the composition of the coral-associated diazotroph community. This work is meaningful for understanding the ecological characteristics of coral-associated diazotrophs and the possible changes in the face of climate change and human activities.

## Materials and Methods

### Study Sites, Coral Sample Collection, and Species Identification

**FIGURE 1:**
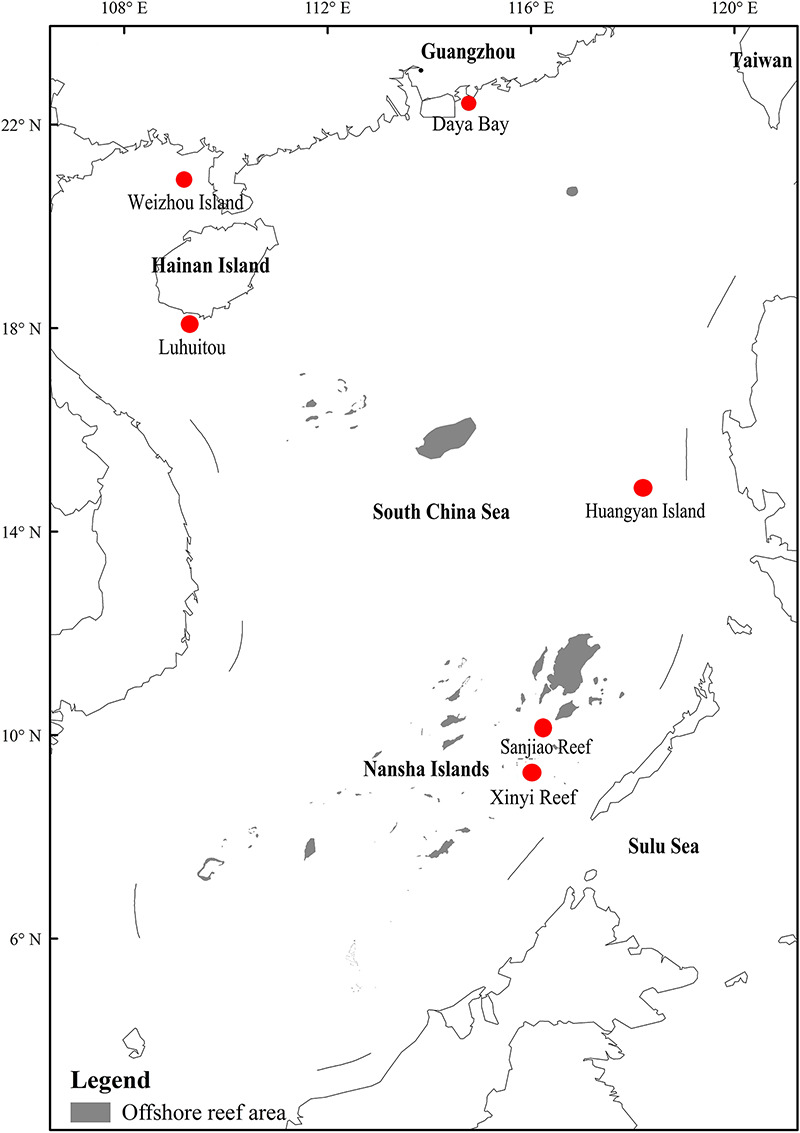
Location map with labeled coral reef areas including six sampling sites in the South China Sea. The map was constructed using ArcGIS software (ver. 10.1). The offshore reef area was drawn using remote sensing images (fusion of Landsat 8 multispectral bands and a panchromatic band) with a resolution of 15 m.

In this study, six locations in the South China Sea, abbreviated as DyB (Daya Bay), HyI (Huangyan Island), Lht (Luhuitou), SjR (Sanjiao Reef), XyR (Xinyi Reef), and WzI (Weizhou Island), were selected (Figure 1). Coral samples were collected using a hammer and chisel by way of scuba diving at a depth of 5–8 m from a specific site in each selected location (Table 1). Three replicate samples (∼6 × 6 cm) were collected from the sides of each colony. The distance between two colonies on the same reef was greater than 10 m. Coral species are represented by the abbreviations Gf (*Galaxea fascicularis*), Gr (*Goniastrea retiformis*), Pd (*Pavona decussate*), Hm (*Hydnophora microconos*), Pl (*Porites lutea*), Pv (*Plesiastrea versipora*), Fp (*Favia palauensis*), Pc (*Plesiastrea curta*), Me (*Montipora efflorescens*), Pe (*Pocillopora eydouxi*), and Ar (*Acropora rosaria*). Not all coral species could be collected from each sampling location. The collected samples were washed with sterile seawater three times and then placed in sterile plastic bags. All samples were briefly stored at low temperatures (0–4°C) and then immediately transported back to the laboratory for DNA extraction.

**TABLE 1:** The locations, coral species, and numbers of sampled corals.

Site location	Sampling date	Coral species	Coral code^#^	Colony number
(longitude, latitude)				
Daya Bay	01 September 2015	*Galaxea fascicularis*	Gf1_DyB, Gf2_DyB	2
(114°38′40″E, 22°34′57″N)		*Goniastrea retiformis*	Gr1_DyB, Gr2_DyB, Gr3_DyB, Gr4_DyB	4
		*Pavona decussata*	Pd1_DyB, Pd2_DyB	2
		*Hydnophora microconos*	Hm1_DyB	1
Weizhou Island	23 October 2015	*Porites lutea*	Pl1_WzI, Pl2_WzI, Pl3_WzI, Pl4_WzI	4
(109°06′40″E, 21°04′30″N)		*Favia palauensis*	Fp1_WzI, Fp2_WzI, Fp3_WzI	3
		*Plesiastrea versipora*	Pv1_WzI, Pv2_WzI, Pv3_WzI	3
Luhuitou	15 October 2016	*Porites lutea*	Pl1_Lht, Pl2_Lht, Pl3_Lht, Pl4_Lht	4
(109°29′16″E, 18°13′18″N)		*Plesiastrea versipora*	Pv1_Lht, Pv2_Lht, Pv3_Lht	3
		*Favia palauensis*	Fp1_Lht	1
Huangyan Island	15 July 2015	*Porites lutea*	Pl1_HyI, Pl2_HyI, Pl3_HyI, Pl4_HyI, Pl5_HyI	5
(117°44′49″E, 15°13′08″N)		*Goniastrea retiformis*	Gr1_HyI, Gr2_HyI, Gr3_HyI	3
		*Plesiastrea versipora*	Pv1_HyI, Pv2_HyI, Pv3_HyI	3
		*Plesiastrea curta*	Pc1_HyI, Pc2_HyI	2
Sanjiao Reef	19 May 2015	*Porites lutea*	Pl1_SjR, Pl2_SjR, Pl3_SjR, Pl4_SjR	4
(115°12′41″E, 10°13′24″N)		*Montipora efflorescens*	Me1_SjR, Me2_SjR	2
		*Pavona decussata*	Pd1_SjR, Pd2_SjR	2
		*Hydnophora microconos*	Hm1_SjR, Hm2_SjR	2
		*Pocillopora eydouxi*	Pe1_SjR	1
		*Favia palauensis*	Fp1_SjR	1
		*Plesiastrea curta*	Pc1_SjR, Pc2_SjR, Pc3_SjR	3
Xinyi Reef	21 May 2016	*Porites lutea*	Pl1_XyR, Pl2_XyR, Pl3_XyR, Pl4_XyR	4
(115°55′49″E, 9°20′06″N)		*Goniastrea retiformis*	Gr1_XyR, Gr2_XyR, Gr3_XyR, Gr4_XyR, Gr5_XyR	5
		*Acropora rosaria*	Ar1_XyR, Ar2_XyR, Ar3_XyR	3
		*Favia palauensis*	Fp1_XyR	1

**^#^The letters before the underscore are the initials of the coral genus and species, which represent the species of corals. The numbers indicate the order of coral individuals. The letters after the underscore represent the abbreviations for the coral reef locations.**

After genomic DNA was extracted, three replicate samples of each colony were merged equally. A total of 68 coral colonies, which included 6 families, 9 genera, and 11 species (Table 1), were identified and selected as the study subjects according to their ecological and morphological characteristics.

### Seawater Collection, Nutrition, and Environmental Factor Detection

Three to five liters of seawater was collected at a depth of 5–8 m using a water sampler around each site. The distance between two sampling sites was not less than 100 m. The temperature (Tem), salinity (Sal), turbidity (Tur), dissolved oxygen (DO), and pH values were immediately measured on site using a thermometer, salinometer, turbidimeter, dissolved oxygen meter, and acidometer, respectively. Pore water was extracted from sediments by centrifugation (3,500 rpm, 40 min), filtered through 0.45 μm-pore-size cellulose acetate filters, and then collected in acid-precleaned vials. Finally, all samples were stored in an icebox for transport to the laboratory and stored in deep freezers (–20°C) until analyses (Guo et al., 2017). All nutrient statuses, including the concentrations of dissolved inorganic nitrogen (DIN; DIN = ${\text{NH}}_{4}^{+}$ + ${\text{NO}}_{2}^{-}$ + ${\mathrm{NO}}_{3}^{-}$), soluble reactive phosphorus (SRP; ${\text{PO}}_{4}^{3-}$, and ${\text{SiO}}_{3}^{2-}$, were measured according to “Specifications for oceanographic survey” (General Administration of Quality Supervision, Inspection and Quarantine of the People’s Republic of China, 1991) (Guo et al., 2017). The average physical and chemical parameters from at least three samples from each coral reef were tested. Longitudes (Lng) and latitudes (Lat) were detected by a global positioning system (GPS). All the data were measured three times and then averaged.

### DNA Extraction, PCR Amplification, and Illumina MiSeq Sequencing

Small pieces of coral samples, including tissue, mucus and skeleton (~50 mg), were cut with a pair of scissors and used for genomic DNA extraction with the TIANamp Marine Animals DNA Kit [Tiangen Biotech (Beijing) Co., Ltd., Beijing, China] according to the manufacturer’s instructions. The nitrogen-fixing gene (*nifH*) of diazotrophs was amplified using the specific forward primer nifH-F (5′-AAAGGYGGWATCGGYAARTCCACCAC-3′) and reverse primer nifH-R (5′-TTGTTSGCSGCRTACATSGCCATCAT-3′), where the barcode was an eight-base sequence unique to each sample (Rösch et al., 2002; Hamady et al., 2008; Mori et al., 2014; Xu et al., 2016). The reaction system and procedure for PCR using an ABI GeneAmp^®^ 9700 thermal cycler and TransGen AP221-02 PCR kit (TransStart FastPfu DNA Polymerase, 20 μL reaction system) were the same as those described in a previous report (Sun et al., 2014). The following steps were employed in the PCR: a 3-min hot start at 95°C after the reaction system was configured according to the manufacturer’s instructions; 35 cycles of denaturation at 95°C for 30 s, annealing at 57°C for 30 s, and elongation at 72°C for 45 s; an extension at 72°C for 10 min; and preservation at 10°C until halted by the user. Triplicate PCR products were pooled for each sample, and fragments with size ranges of 421–440 bp were then purified and quantified using an AxyPrep DNA gel extraction kit (Axygen Biosciences, Union City, CA, United States) and QuantiFluor™-ST fluorometer (Promega, United States). Purified amplicons were pooled in equimolar amounts and paired-end sequenced (2 × 250 bp) on the Illumina MiSeq platform according to standard protocols (Majorbio Bio-Pharm Technology Co., Ltd., Shanghai, China). The datasets analyzed during the current study are available at the NCBI Sequence Read Archive repository under accession number SRP145254^1^.

### Data Analysis

Raw sequences were demultiplexed, quality-filtered by Trimmomatic, and merged by FLASH with the following criteria (Magoč and Salzberg, 2011): (i) The reads were truncated at any site receiving an average quality score <20 over a 50 bp sliding window (Bolger et al., 2014). (ii) Primers were exactly matched allowing two nucleotide mismatching, and reads containing ambiguous bases were removed. (iii) Sequences whose overlap longer than 10 bp were merged according to their overlap sequence. The merged sequences were clustered into operational taxonomic units (OTUs) with a 97% similarity cutoff (Dang et al., 2018) using UPARSE software (version 7.1)^2^ and chimeric sequences were then identified and removed using UCHIME software (Edgar, 2010). The taxonomy of representative sequences was analyzed by the RDP Classifier algorithm^3^ against the fgr/nifH database (release 7.3)^4^ using confidence threshold of 70% (Edgar, 2010). The OTUs were then analyzed: alpha diversity index were estimated using mothur (version v.1.30.1) (Schloss et al., 2011), and beta diversity analyze and other analyses were calculated using QIIME and R packages.

In the present study, the rarefaction curve basing on sobs index was used to assessment whether the sequencing depth is enough for each sample. ACE was used to estimate community richness and the larger value indicates the higher richness. Shannon was used to estimate community diversity and the larger value indicates the higher diversity. The calculation formulas for ACE and Shannon could be viewed on this website^5^.

The differences in the indices between each group of samples were tested using Student’s *t*-test. Significance was declared at *P* ≤ 0.05. The taxonomy was assigned and compared with that in the fgr/nifH database (Quast et al., 2013) using the QIIME platform^6^. Moreover, similarities or differences in the composition of bacterial communities were reflected by principle coordinate analysis (PCoA) using Bray-Curtis distances at the OTU level (Lozupone and Knight, 2005). A heatmap of the correlations between environmental variables and nitrogen-fixing bacteria associated with corals was constructed by Spearman’s correlation test and GraphPad Prism version 6.00 (GraphPad Software, San Diego, CA, United States). Significance was assigned at *P* ≤ 0.05.

## Results

### Nutrient Parameters of Sampling Sites

Six sampling locations were distributed across different areas in the South China Sea. The results of the analysis in Table 2 indicate that concentrations of DIN and other nutrients were very high (3.17 μmol/L ≥ DIN ≥ 2.31 μmol/L, 29.75 μmol/L ≥ SiO_3_^2–^ ≥ 17.25 μmol/L, and 0.51 μmol/L ≥ SRP ≥ 0.38 μmol/L) in some coastal sampling locations (e.g., the high-latitude DyB, WzI, and Lht sites). In contrast, the concentrations of these nutrients were relatively low in the low-latitude island reefs at HyI, XyR, and SjR, which were far from land (1.42 μmol/L ≥ DIN ≥ 1.27 μmol/L, 2.40 μmol/L ≥ SiO_3_^2–^ ≥ 1.62 μmol/L, and 0.07 μmol/L ≥ SRP ≥ 0.03 μmol/L). The turbidity was also significantly higher at the fringing reefs than at the island reefs (3.25 NTU ≥ Tur ≥ 1.16 NTU and 0.4 NTU ≥ Tur ≥ 0.2 NTU, respectively). The high-latitude DyB, WzI, and Lht sites were constantly disturbed by human activity, while the low-latitude HyI, XyR, and SjR sites were less disturbed. This difference was the cause of the difference in nutrient distribution.

**TABLE 2:** Nutrient parameters from different sampling sites in the South China Sea.

Index	DyB	WzI	Lht	HyI	XyR	SjR
DO (mg/L)	7.18	6.67	7.22	6.88	7.29	7.24
Tem (°C)	28.00	27.40	29.00	30.90	31.1	30.81
pH	8.20	8.38	8.29	8.42	8.28	8.10
Sal (%)	3.35	3.26	3.31	3.38	3.34	3.33
DIN (μmol/L)	3.17	2.67	2.31	1.27	1.27	1.42
SRP (μmol/L)	0.38	0.51	0.45	0.07	0.03	0.04
SiO_3_^2–^ (μmol/L)	20.21	29.75	17.25	2.57	1.62	2.40
Lng (°)	114.60	109.10	109.90	117.70	115.90	115.20
Lat (°)	22.60	21.07	20.20	15.20	9.30	10.20
Tur (NTU)	1.16	1.30	3.25	0.20	0.40	0.30

### Diversity of Coral-Associated Diazotrophs

**FIGURE 2:**
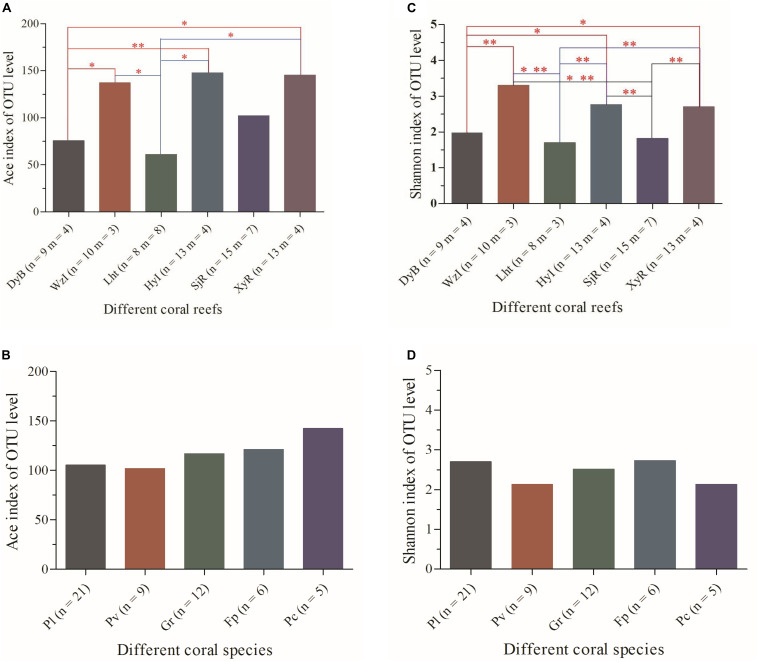
Analysis of the differences in alpha-diversity indices of coral-associated diazotrophs between sample groups at the OTU level. **(A)** ACE between coral reefs, **(B)** ACE between coral species, **(C)** Shannon index between coral reefs, and **(D)** Shannon index between coral species. The analysis used was Student’s *t*-test. Significant differences are indicated by different numbers of asterisks (0.01 < *p* ≤ 0.05 ^∗^, 0.001 < *p* ≤ 0.01 ^∗∗^). Non-significant correlations do not have an asterisk. *n*, number of coral individuals; *m*, number of coral species.

A total of 1,223,398 reads recovered from 68 coral samples, with lengths ranging from 421 to 440 bp, were obtained from the sequencing database. Good’s coverage of each sequencing database was greater than 99% (Supplementary Table S1 and Supplementary Figure S1). Thus, these sequencing results accurately represented the diazotrophs in the coral samples. Other indices, including the abundance-based coverage estimator (ACE) and Shannon index, are shown more intuitively in Figure 2. The detailed data showed that the ACE, which reflects community richness, varied greatly among coral samples (Supplementary Table S1). The lowest values were 21.35, 30.79, and 31.54 from Pv3_Lht, Pl1_SjR, and Pl2_SjR, respectively, while the highest values were 300.4, 293.57, and 286.85 from Gr3_XyR, Fp3_WzI, and Ar3_XyR, respectively. The Shannon index (Supplementary Table S1), which reflects community diversity, also differed significantly among coral samples (ranging from 0.58 to 4.48). When coral individuals were grouped according to different species and sampling regions, these indices showed non-significant differences between most species at *P* > 0.05 but significant differences between most sampling regions at *P* ≤ 0.05 (Figure 2). The average ACE values for WzI, HyI, XyR, and SjR (146.99, 139.83, 145.23, and 102.12, respectively) were obviously higher than those for Lht and DyB (71.07 and 75.54, respectively). Meanwhile, the average Shannon values for WzI, HyI, and XyR (3.34, 2.76, and 2.70, respectively) were also higher than those for DyB, SjR, and Lht (1.98, 1.82, and 1.70, respectively). However, the ACE and Shannon index averages for different coral species ranged from 101.73 to 150.28 and 2.12 to 2.73, respectively, with small ranges of fluctuations between them (Figures 2B,D). These findings suggest that geographical factors have a strong effect on the community richness and diversity of coral-associated diazotrophs.

The number of OTUs and the diversity at various taxonomic levels are listed in Supplementary Table S2. The results showed great differences in the number of communities of diazotrophs between coral individuals, even for the same coral species in the same sampled location. For example, 6 phyla, 21 genera, and 280 OTUs were detected in Gr3_XyR, but the numbers were 5, 11, and 50 in Gr1_XyR, respectively.

### Clustering of Coral-Associated Diazotrophs Based on Similarity

**FIGURE 3:**
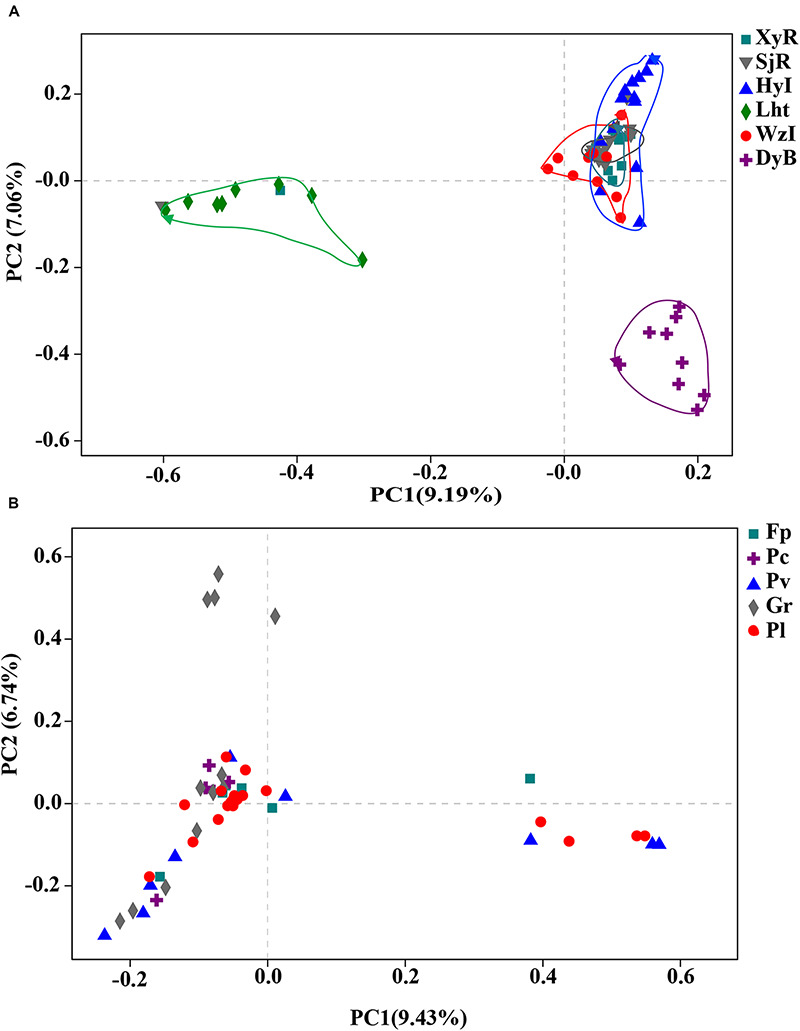
PCoA plot at the OTU level for all coral samples collected from six different coral reefs. Coral samples were grouped according to **(A)** collection site and/or **(B)** species with ≥5 colonies from the same coral reef. Scatter plot showing principal coordinate 1 (PC1) vs. principal coordinate 2 (PC2). PC1 and PC2 represent the principal factors affecting the composition of coral-associated diazotrophs.

The similarity among the diazotrophic communities associated with the 68 coral samples from 6 locations was evaluated using PCoA at the OTU level. The diazotrophic composition of coral individuals differed between some coral reefs, regardless of whether the coral individuals were of the same species (Figure 3A). Coral individuals from Lht and DyB were clustered together differently from the other four reef areas (WzI, HyI, XyR, and SjR). This difference may be related to the surrounding seawater environment. Lht and DyB are high-latitude fringing reefs, and the other sites are low-latitude island reefs (except WzI, which is a high-latitude fringing reef). In comparison with the grouping of regions, coral individuals from the same species did not cluster together (Figure 3B). This results indicated that the diazotrophic composition associated with corals was somewhat species specific. Overall, the key factor affecting diazotrophic composition was geographical position, rather than interspecific differences.

### Composition of Diazotrophs Associated With Corals

**FIGURE 4:**
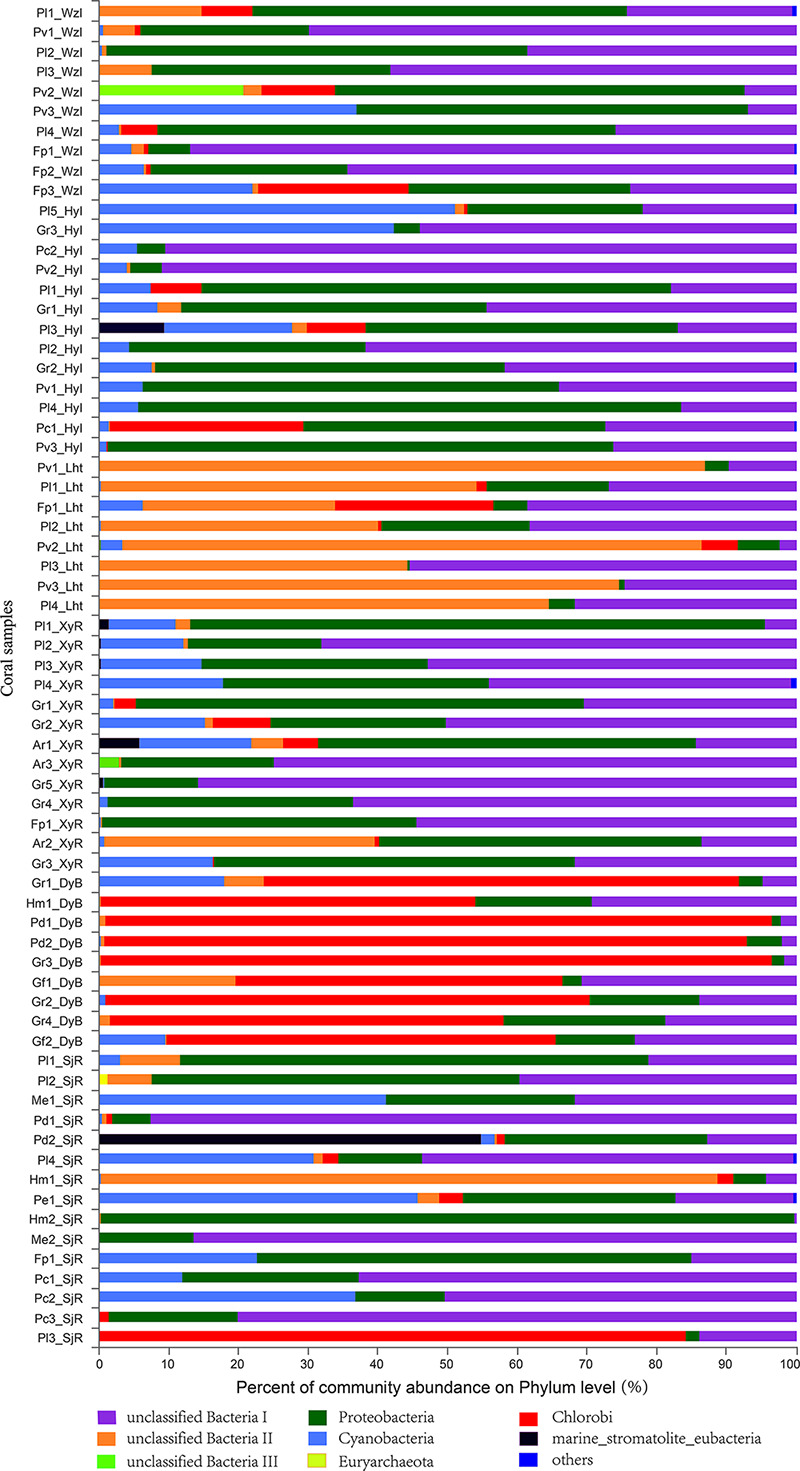
Composition profiles of diazotrophs. Taxonomic classification of bacterial reads retrieved from all coral samples at the phylum level using RDP Classifier.

Eleven bacterial phyla capable of nitrogen fixation, including three unclassified bacteria (I, II, and III), were identified from the sequencing database of 68 coral samples (Figure 4). Among these phyla, unclassified bacteria I, which had a very high relative abundance, was present in almost all the coral samples. In addition, the dominant bacterial phyla were Proteobacteria, Chlorobi, and Cyanobacteria. The relative abundance of these coral-associated bacterial phyla exhibited significant differences between sites. For example, unclassified bacteria I and Proteobacteria were the dominant bacterial phyla (>80% relative abundance) in most of the coral samples from WzI, HyI, XyR, and SjR. In contrast, the relative abundance of these two bacterial phyla was low (<35%) in most of the coral samples from Lht and DyB. In particular, the relative abundance of Proteobacteria was generally very low (<10%) in most of the coral samples from these two locations; the dominant diazotrophs in these locations were unclassified bacteria II (Lht) and Chlorobi (DyB). Of course, there were special cases. For example, the relative abundance of Chlorobi, which was the dominant group, was 84% in Pl3_SjR from SjR. Unclassified group II was the dominant group, with a relative abundance of 89%, in Hm1_SjR from SjR. Cyanobacteria, members of which are capable of nitrogen fixation, was also a common bacterial phylum in these coral samples. The relative abundance of Cyanobacteria ranged from 0.21% (Pl2_SjR, Pl3_Lht, Pv3_Lht, etc.) to 51% (Pl5_HyI). The other nitrogen-fixing groups, including Euryarchaeota, Firmicutes, and Verrucomicrobia, were detected in a small number of coral samples and had very low relative abundance (except marine stromatolite eubacteria in Pd2_SjR). At the class level (Supplementary Figure S2), the dominant taxa were unclassified_p_unclassified bacteria I and unclassified Proteobacteria in most coral samples from WzI, HyI, XyR, and SjR. Chlorobi was the dominant class in all coral samples from DyB (ranging from 47 to 96%) and Pl3_SjR (84%) from SjR. In addition, unclassified_p_unclassified bacteria II was the dominant group in most coral samples from Lht (ranging from 28 to 87%) and Hm1_SjR from SjR (89%). The relative abundance of most other classes, including Alphaproteobacteria, Betaproteobacteria, and Deltaproteobacteria, was very low. At other taxonomic levels (order, family, genus, and species), the unclassified diazotrophs were the dominant groups. These results indicated that the coral holobionts contained many diazotrophs that have not been isolated and recognized.

**FIGURE 5:**
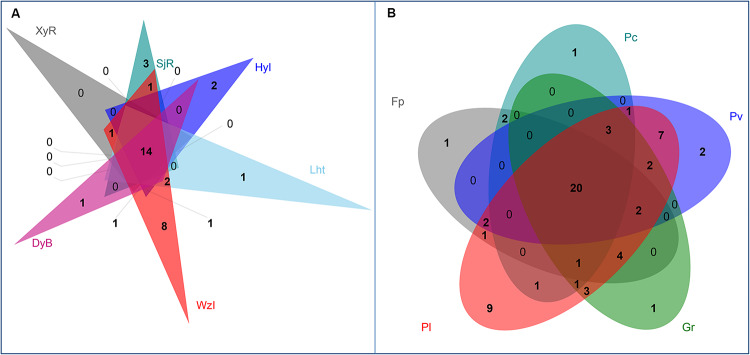
Venn diagrams showing the number of diazotrophs at the genus level from different coral reefs **(A)** and coral species **(B)**.

At the genus level, Venn diagrams showed that most diazotrophic genera were common (14 and 20 among coral species and sampling regions, respectively) in multiple sample groups, which were core members (Figure 5). Among these genera, *Vibrio* and *Chlorobium* overlapped exactly among coral reefs and coral species (Table 3). In addition to the overlapping genera, there were some unique diazotrophic genera for each sample group, whether in coral species or sampling regions (Table 4). For example, *Desulfobacter* was restricted to coral individuals from DyB. *Chroococcidiopsis* appeared only in the coral species *Goniastrea retiformis*. Overall, 15 diazotrophic genera were restricted to different groups of sampling regions, and 14 genera were specific to different groups of coral species.

**TABLE 3:** Overlapping diazotrophic genera in different sample groups.

Bacterial genera in different coral reefs	Bacterial genera in different coral species
	[Pl (*n* = 21), Pv (*n* = 9), Gr (*n* = 12), Fp (*n* = 6), and Pc (*n* = 5)]
g_unclassified_f_Chlorobiaceae g_*Teredinibacter* g_*Vibrio* g_norank_c_unclassified_Cyanobacteria g_unclassified_c_Alphaproteobacteria g_unclassified_c_norank_p_Cyanobacteria g_unclassified_k_norank_d_Bacteria g_unclassified_p_Cyanobacteria g_unclassified_o_Rhizobiales g_unclassified_p_Proteobacteria g_unclassified_c_Gammaproteobacteria g_unclassified_p_unclassified Bacteria II g_*Chlorobium* g_unclassified_c_Deltaproteobacteria	g_unclassified_o_Rhizobiales g_*Vibrio* g_unclassified_o_Desulfuromonadales g_norank_c_unclassified_Cyanobacteria g_unclassified_c_Alphaproteobacteria g_unclassified_c_norank_p_Cyanobacteria g_unclassified_k_norank_d_Bacteria g_unclassified_ o_Chroococcales g_*Desulfuromonas* g_*Mastigocoleus* g_unclassified_f_Chromatiaceae g_unclassified_p_Cyanobacteria g__unclassified_f__Chlorobiaceae g__unclassified_o__Chromatiales g_unclassified_p_Proteobacteria g_unclassified_c_Gammaproteobacteria g_unclassified_p_unclassified Bacteria II g_*Bradyrhizobium* g_*Chlorobium* g_unclassified_c_Deltaproteobacteria

**n, number of coral individuals; m, number of coral species. The red-labeled bacteria are the species with exact overlap between coral reefs and coral species.**

**TABLE 4:** Specific diazotrophic genera in different sample groups.

Coral reefs	Bacterial genera	Coral species	Bacterial genera
DyB (*n* = 9, *m* = 4)	g_*Desulfobacter*	Pl (*n* = 21)	g_*Stenotrophomonas* g_*Zoogloea* g_norank_p_unclassified bacteria g_norank_f_ Deltaproteobacteria g_unclassified_c_Clostridia g_*Rhodospirillum* g_*Tolumonas* g_unclassified_p_Euryarchaeota g_unclassified_f_Desulfobacteraceae
WzI (*n* = 10, *m* = 3)	g_*Calothrix* g_*Stenotrophomonas* g_unclassified_f_Desulfobacteraceae g_norank_p_unclassified bacteria g_norank_f_ Deltaproteobacteria g_*Klebsiella* g_unclassified_f_Rhodocyclaceae g_*Rhodospirillum*		
Lht (*n* = 8, *m* = 3)	g_*Azotobacter*	Pv (*n* = 9)	g_*Klebsiella* g_unclassified_f_Rhodocyclaceae
HyI (*n* = 13, *m* = 4)	g_*Desulfarculus* g_unclassified_o_Clostridiales	Gr (*n* = 12)	g_*Chroococcidiopsis*
		Fp (*n* = 6)	g_*Skermanella*
SjR (*n* = 15, *m* = 7)	g_*Rhizobium* g_unclassified_c_Clostridia g_unclassified_p_Euryarchaeota	Pc (*n* = 5)	g_unclassified_o_Clostridiales
XyR (*n* = 13, *m* = 4)	None		

**The indications for “n” and “m” are consistent with those in*Table 3.*

### Environmental Variables Affecting the Distribution of Diazotrophic Species

**FIGURE 6:**
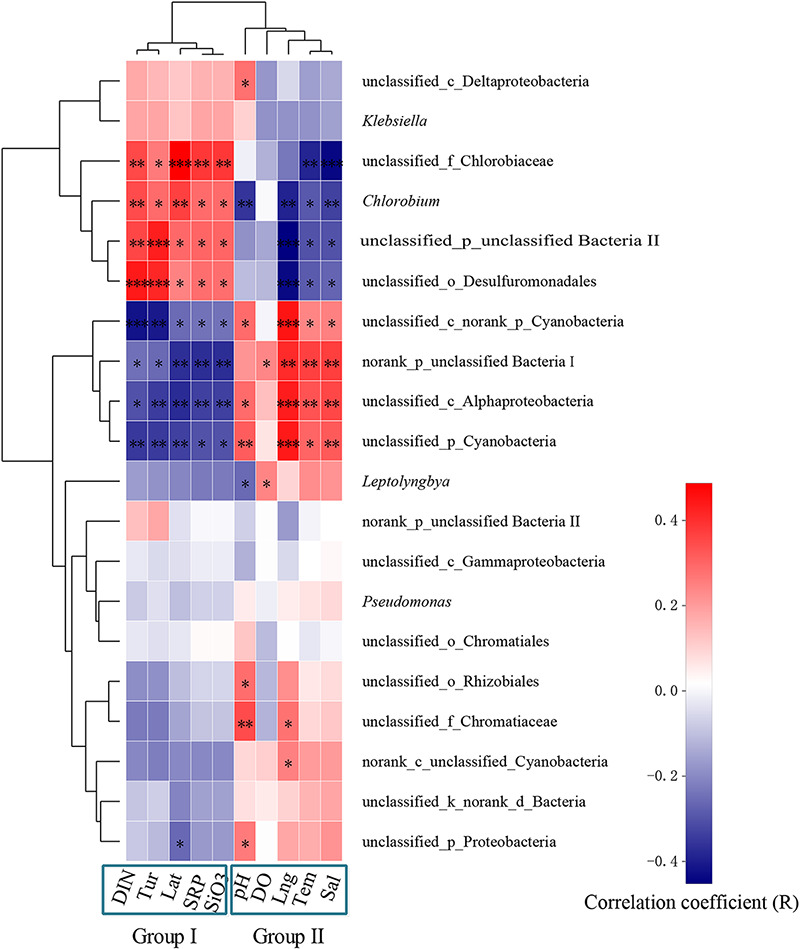
Correlation analyses between environmental parameters and populations of diazotrophs at the genus level. The diazotrophs analyzed were the top 20 genera in terms of total abundance. Hierarchical clustering of environmental variables was performed based on the raw data, while diazotrophic species were clustered based on averages. Significant differences are indicated by different numbers of asterisks (0.01 < *p* ≤ 0.05^∗^, 0.001 < *p* ≤ 0.01^∗∗^, *p* ≤ 0.001^∗∗∗^). Non-significant correlations do not have an asterisk. The R value represents the correlation coefficient, and the closer it is to 1, the more significant the correlation is.

The six sampling locations were distributed in different areas of the South China Sea. These coral reefs were affected by different environmental factors due to their geographical locations (Table 2). The concentrations of DIN and other nutrients were lower in HyI, XyR, and SjR, which were far from land, than in the other locations. In contrast, these nutrient concentrations were 2–10 times higher in some coastal sampling locations (e.g., DyB, WzI, and Lht) than in those far from land. Tur was also significantly higher in the coastal reefs than in the offshore reefs. The correlations between various environmental parameters and communities of coral-associated diazotrophs (the top 20 taxa based on total abundance at the genus level) were then evaluated (Figure 6). The results showed that the effects of different environmental factors on bacterial communities were significantly different. According to similar effects, environmental factors were clustered into two groups: group I, including DIN, Tur, Lat, SRP, and SiO_3_^2–^, and group II, including pH, DO, Lng, Tem, and Sal. Group I was positively correlated with unclassified_f_Chlorobiaceae (0.26 ≤ *R* ≤ 0.49), *Chlorobium* (0.29 ≤ *R* ≤ 0.37), unclassified_p_unclassified Bacteria II (0.30 ≤ *R* ≤ 0.43) and unclassified_o_Desulfuromonadales (0.25 ≤ *R* ≤ 0.44) and negatively correlated with four other bacterial genera, namely, unclassified_c_norank_p_Cyanobacteria (−0.42 ≤ *R* ≤ −0.24), norank_p_unclassified Bacteria I (−0.37 ≤ *R* ≤ −0.25), unclassified_c_Alphaproteobacteria (−0.44 ≤ *R* ≤ −0.30), and unclassified_p_Cyanobacteria (−0.44 ≤ *R* ≤ −0.30). The correlations of other environmental factors with a series of bacterial genera in group II were generally opposite to those in group I (Figure 6).

## Discussion

Microbial nitrogen fixation requires a nitrogenase gene, *nifH*, which has been confirmed to be consistent with the phylogenesis of the 16S rRNA gene (Zehr et al., 2003). Our annotation results revealed that microorganisms capable of nitrogen fixation were ubiquitously present in coral holobionts. Their presence was not related to the external environment. The six sampling locations were distributed in different areas of the South China Sea. The concentrations of DIN and other nutrients were very high at some fringing reef sampling locations (e.g., DyB, WzI, and Lht). In contrast, the concentrations of these nutrients were low in some island reefs (HyI, XyR, and SjR) that were far from land. It is possible that the nutrients in coastal coral reefs could completely meet the nitrogen demands of coral holobionts, even without biological nitrogen fixation. There was evidence that the coral had established symbiotic relationships with some bacteria (including diazotrophs) in their early life history stages, and the bacterial communities then transitioned into a state of long-term dynamic change (Lema et al., 2014). This finding is a reflection of the response of coral holobionts to environmental changes. It can also be speculated that coral-associated diazotrophs have many other biological functions in addition to nitrogen fixation, such as the coordination of carbon fixation. For example, Cyanobacteria is a phylum of prokaryotic microorganisms that can carry out photosynthesis with oxygen production (Hamilton et al., 2016). They can also fix atmospheric nitrogen and convert it into ammonia (NH_3_), nitrite (${\text{NO}}_{2}^{-}$), or nitrate (${\text{NO}}_{3}^{-}$), which can provide available nitrogen to other organisms (Lesser et al., 2004; Charpy et al., 2010).

In this study, the most striking finding was that the community composition of coral-associated diazotrophs, which was identified via the analysis of alpha- and beta-diversity and species differences, was highly significantly different between sampling regions (Figure 2). Notably, community richness, which was reflected by the ACE, was lower for the coral-associated diazotrophs from DyB and Lht than for those from WzI, HyI, SjR, and XyR (Figure 2A). This difference may be attributed to the reef types of DyB and Lht, which are typical fringing reefs that are frequently affected by human activities and contain high concentrations of nutrients and DO. The sites with low community diversity, which was reflected by the Shannon index, exhibited the same trend as the ACE for the coral-associated diazotrophs from DyB and Lht (Figure 2C). This finding may be related to the fact that high concentrations of nutrients (especially DIN) can meet some of the nitrogen requirements of corals. However, this finding was contrary to the results of the analysis of the coral-associated bacterial diversity obtained by high-throughput sequencing based on 16S rRNA gene amplification. Li et al. (2013) studied the bacterial diversity associated with *P. lutea*, *G. fascicularis*, and *Acropora millepora* sampled from Lht. The results showed that the ACE and Shannon index were 855.53–8970.90 and 4.16–7.04, respectively. Additionally, our previous study found that the ACE and Shannon index values of 25 scleractinian coral samples from XyR were 332.22–1500.66 and 1.91–5.88, respectively (Liang et al., 2017). McKew et al. (2012) also showed that the bacterial communities from Caribbean corals were significantly more diverse than those from Indonesian corals.

In fact, the relationships between coral-associated bacteria were structured by multiple factors at different scales. For example, the diversity and composition of the bacterial communities associated with corals are significantly affected by various factors, including coral species (Hong et al., 2009), geography (McKew et al., 2012), skeletal morphology (Liang et al., 2017), and others (Bourne et al., 2008; Ceh et al., 2012). However, the relationship between coral-associated nitrogen-fixing bacteria and other bacteria is not yet clear. In this study, the characteristics of the dominant coral-associated diazotrophs, which exhibited significant geographical differences, were clearly recognized. At the same time, some core diazotrophs were found in different coral reefs or coral species. In addition, there were some unique diazotrophs in different coral reefs or coral species. The PCoA also showed that coral samples from different locations were clearly separated based on their diazotrophic composition (Figure 3). We speculate that the selection of coral-associated diazotrophs is directly related to the environmental factors of the surrounding seawater. Because the environmental variables significantly differed between these coral reefs, correlation analysis revealed that various environmental factors were positively or negatively correlated with different bacterial genera (Figure 6). For example, the effects of group I (Lat, SRP, SiO_3_^2–^, DIN, and Tur) and group II (pH, DO, Tem, Sal, and Lng) on most of the diazotrophic communities were similar. The correlations between the distribution of nitrogen-fixing bacteria associated with corals and environmental factors were reported for the first time in this study. The findings of this study shed light on the communities and dominant groups of diazotrophs in characteristic coral reefs and their relationships with key environmental variables.

In summary, our results fully reflected the diversity of diazotrophs associated with different coral species sampled from several coral reefs that exhibited differences in environmental variables in the South China Sea. Although many diazotrophic species were unclassified, it was shown that the predominant taxa of diazotrophs among six different coral reefs exhibited more significant geographical differences than interspecific differences. In addition, the correlation analysis revealed that various environmental factors were positively or negatively correlated with different bacterial genera. We believe that corals tend to be associated with unclassified_f_Chlorobiaceae, *Chlorobium*, unclassified_p_unclassified Bacteria II, and unclassified_o_Desulfuromonadales as symbiotic nitrogen-fixing bacteria under changing nutrient enrichment conditions. In addition, corals from high-latitude reefs may tend to be associated with unclassified_c_norank_ p_Cyanobacteria, norank_p_unclassified Bacteria I, unclassified_ c_Alphaproteobacteria, and unclassified_p_Cyanobacteria under the effects of global warming. This finding occurs because with the influence of global warming and human activities, the environmental stress on global coral reefs is becoming increasingly serious. In the South China Sea, coral reefs at low latitudes are also often subjected to unusually high temperatures. Statistics show that the Nansha Islands (where XyR and SjR are located) had annual maximum temperatures between 30 and 31°C from 1985 to 2015 (Qin et al., 2019). The coral reefs located in high-latitude regions (e.g., Lht and DyB) are affected by human activities all year, resulting in serious eutrophication of the surrounding seawater. The live coral cover rapidly decreased from greater than 60% to less than 20% from 1984 to 2015 (Zhao et al., 2012; Qin et al., 2019). Diazotrophs are essential components of coral holobionts, which are involved in the nitrogen cycle (Piniak et al., 2003; Olson et al., 2009) and are important for energy supply (Hamilton et al., 2016). There was a significant positive correlation between four diazotrophic bacterial genera (unclassified_c_norank_p_Cyanobacteria, norank_p _unclassified Bacteria I, unclassified_c_Alphaproteobacteria, and unclassified_p_Cyanobacteria) and seawater temperature (0.001 < *p* ≤ 0.05). Therefore, we believe that with global warming, functional nitrogen-fixing bacteria in corals will gradually evolve to be dominated by genera such as Cyanobacteria and Alphaproteobacteria to adapt to increasing sea surface temperatures, especially in low-latitude coral reefs such as SjR and XyR. In addition, although the number of living corals is decreasing under high nutrient stress, some individuals with environmental tolerance can survive. This phenomenon is closely related to the adaptability of symbiotic microorganisms in coral. Our results showed that four diazotrophic bacterial genera (unclassified_f_Chlorobiaceae, *Chlorobium*, unclassified_p_unclassified Bacteria II, and unclassified_o_Desulfuromonadales) were positively correlated with the concentrations of nutrients such as DIN, SRP, and SiO_3_^2–^ (*p* ≤ 0.05). It is possible that genera such as Chlorobiaceae, *Chlorobium*, and Desulfuromonadales will become the dominant diazotroph groups in coral if the surrounding eutrophication continues to deteriorate at high-latitude fringing reefs (e.g., Lht and DyB). In fact, the results of the diazotroph composition also verified this conjecture (Figure 4). In particular, the relative abundance of Chlorobi was very high at the high-latitude DyB site where the annual average temperature was low. This finding may be related to the photosynthetic energy demand of coral holobionts. The effective photochemical efficiency provided by *Symbiodinium* is relatively weak under low temperature conditions (Xu et al., 2017). Chlorobi may not only perform the function of nitrogen fixation but also supplement photosynthetic energy. An understanding of the diazotrophic communities associated with scleractinian corals from different reef areas will help us to understand that the evolution of microbial populations with specific functions, which represent a strategy for coral hosts to adapt to environmental changes.

## Data Availability Statement

The datasets analyzed during the current study are available from the [NCBI Sequence Read Archive] repository under accession number SRP145254 [https://www.ncbi.nlm.nih.gov/search/all/?term=SRP145254].

## Ethics Statement

Permits for coral sampling were provided by the State Oceanic Administration, People’s Republic of China, and the local Department of Ocean and Fisheries.

## Author Contributions

KY and JL conceived the research and wrote the manuscript. YW, XH, WH, and ZW contributed to the materials. JL performed all the experiments. ZQ and GW constructed all the figures. BC and HS identified coral species. All authors edited and approved the manuscript.

## Conflict of Interest

The authors declare that the research was conducted in the absence of any commercial or financial relationships that could be construed as a potential conflict of interest.

## Footnotes

1 https://www.ncbi.nlm.nih.gov/search/all/?term=SRP145254

2 http://drive5.com/uparse/

3 http://rdp.cme.msu.edu/

4 http://fungene.cme.msu.edu/nifH

5 http://www.mothur.org/wiki/Calculators

6 http://qiime.org/scripts/assign_taxonomy.html
