# Supplementary material for: Diazotroph Diversity Associated With Scleractinian Corals and Its Relationships With Environmental Variables in the South China Sea
Source: Front Physiol. 2020 Jun 17;11:615. doi: 10.3389/fphys.2020.00615 (PMC7314963; doi:10.3389/fphys.2020.00615)
Supplement: Supplementary file 1 [file Datasheet_1.doc]

**Diazotroph Diversity Associated with Scleractinian Corals and Its Relationships with Environmental Variables in the South China Sea**

Jiayuan Liang^1,2,3^, Kefu Yu^1,2,3*^, Yinghui Wang^1,2,3^, Xueyong Huang^1,2,3^, Wen Huang^1,2,3^, Zhenjun Qin^1,2,3^, Guanghua Wang^1,2,3^, Hongfei Su^1,2,3^, Biao Chen^1,2,3^, Zhengchao Wu^4^

^*^Corresponding author, E-mail address: kefuyu@scsio.ac.cn (K. Yu), Tel & Fax: +86-771-3231358, ORCID number: 0000-0003-3409-9945

^1^Coral Reef Research Center of China, Guangxi University, Nanning 530004, China, ^2^Guangxi Laboratory on the Study of Coral Reefs in the South China Sea, Nanning 530004, China, ^3^School of Marine Sciences, Guangxi University, Nanning 530004, China, ^4^State Key Laboratory of Tropical Oceanography (LTO), South China Sea Institute of Oceanology, Chinese Academy of Sciences, Guangzhou 510301, China

**Figure S1** Rarefaction curve of each sample. The same color represented the same coral species (Pl, Pv, Gr, Fp, Pd, Pc, and Ar).

**
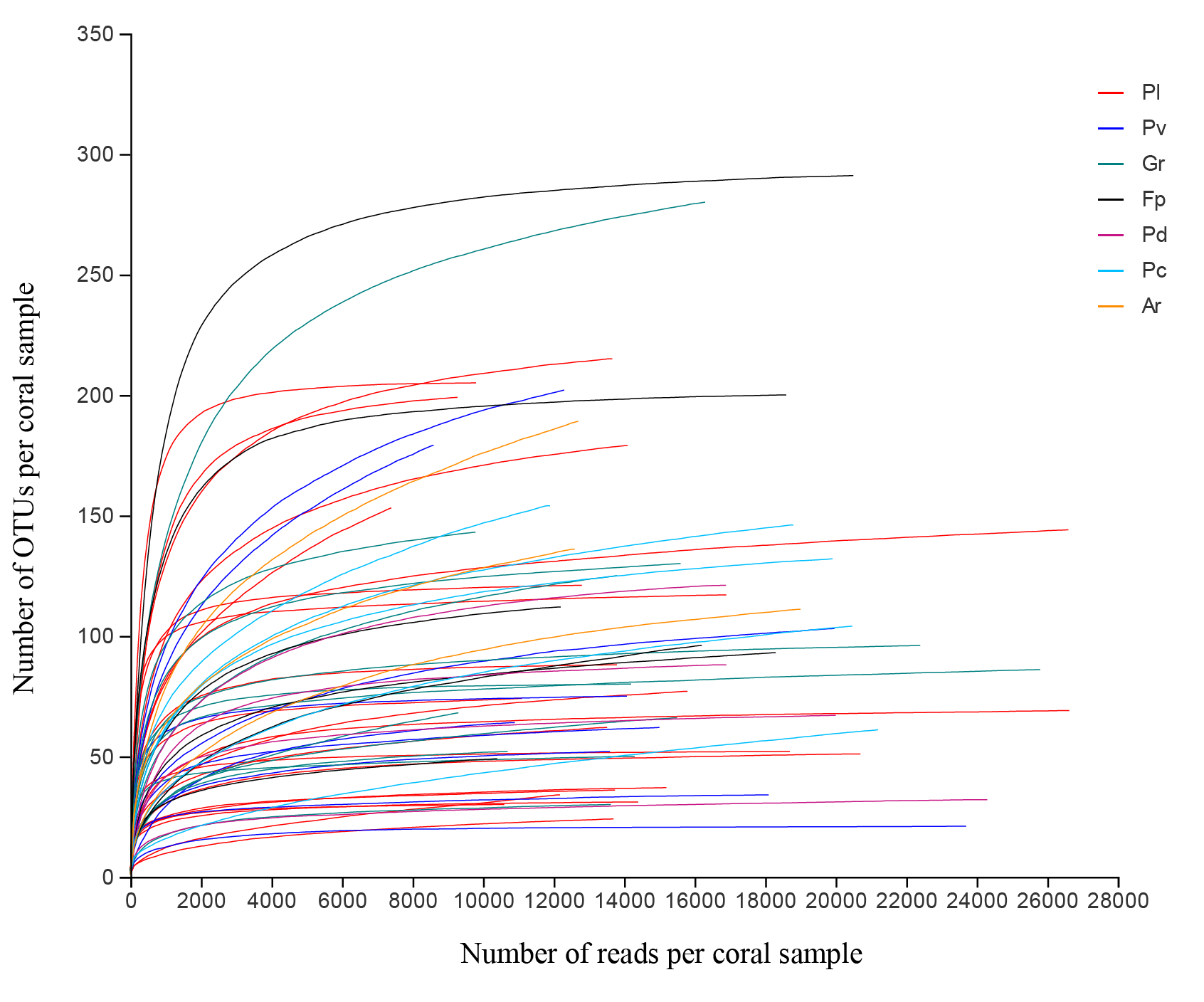
**

**Figure S2** Composition profiles of diazotrophs. Taxonomic classification of bacterial reads retrieved from all the coral samples at the class level using RDP Classifier; “others” represents the bacteria with an abundance less than 0.1%.

**
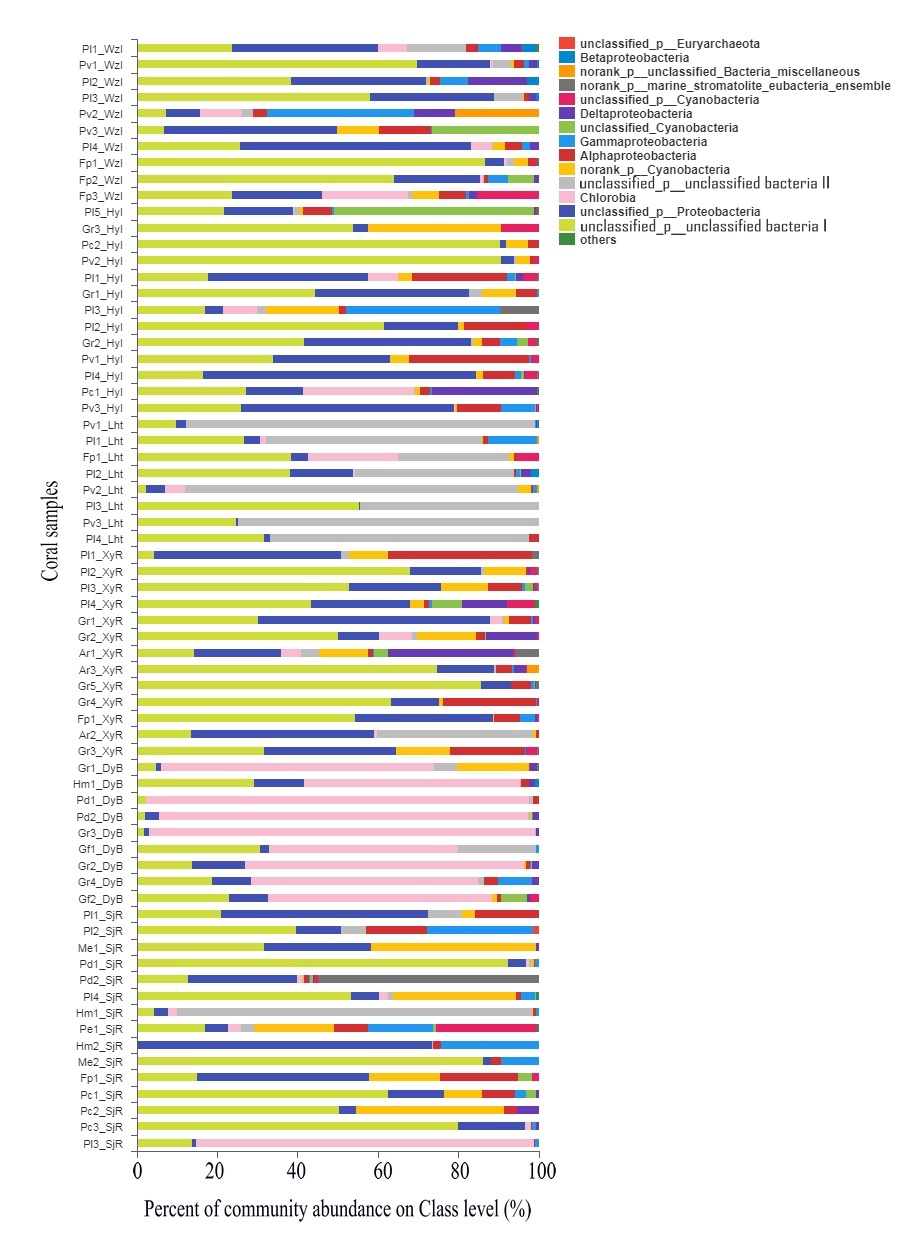
**

**Table S1** Number of sequences and diversity estimates of diazotrophs in different coral colonies.

| Coral code^#^ | No. of Seq. | ACE | Shannon index | Coverage |
| --- | --- | --- | --- | --- |
| Gr3_HyI | 13285 | 141.17 | 2.00 | 0.997413 |
| Pv1_Lht | 11545 | 70.18 | 1.24 | 0.998982 |
| Pv3_HyI | 17471 | 233.54 | 3.46 | 0.996828 |
| Pl4_HyI | 18426 | 192.95 | 3.23 | 0.998508 |
| Pv2_WzI | 18633 | 37.02 | 2.10 | 0.999833 |
| Pc1_SjR | 22940 | 173.42 | 2.55 | 0.998289 |
| Gr2_XyR | 18131 | 139.06 | 3.23 | 0.999165 |
| Pv3_Lht | 24161 | 21.35 | 0.90 | 0.999958 |
| Gf2_DyB | 23738 | 148.30 | 3.13 | 0.999634 |
| Pv3_WzI | 23322 | 115.06 | 2.50 | 0.999096 |
| Pl1_XyR | 11611 | 77.85 | 3.30 | 0.999457 |
| Pd2_DyB | 18406 | 91.92 | 1.39 | 0.999586 |
| Pl4_WzI | 17766 | 222.85 | 4.06 | 0.998679 |
| Hm1_DyB | 16361 | 50.33 | 2.33 | 0.999934 |
| Gr4_XyR | 17135 | 95.66 | 1.84 | 0.998899 |
| Gr1_DyB | 10963 | 57.54 | 1.35 | 0.999343 |
| Pl3_XyR | 12964 | 256.71 | 3.17 | 0.993493 |
| Pc3_SjR | 21942 | 144.46 | 1.88 | 0.999045 |
| Fp1_SjR | 19140 | 128.71 | 2.17 | 0.998146 |
| Pl3_WzI | 15364 | 88.96 | 3.15 | 0.999782 |
| Me1_SjR | 20853 | 192.13 | 2.32 | 0.997458 |
| Gr1_XyR | 14815 | 58.06 | 2.75 | 0.99972 |
| Gr3_XyR | 22296 | 300.40 | 4.02 | 0.997791 |
| Pl2_WzI | 18142 | 121.75 | 4.35 | 0.999704 |
| Fp1_XyR | 14126 | 119.59 | 2.37 | 0.998933 |
| Fp1_WzI | 19497 | 106.87 | 1.47 | 0.999122 |
| Pl1_WzI | 12407 | 205.20 | 4.48 | 0.999898 |
| Pl4_XyR | 20039 | 52.35 | 3.09 | 0.999946 |
| Pv1_WzI | 14817 | 77.03 | 2.77 | 0.999715 |
| Pd1_DyB | 24644 | 42.18 | 1.56 | 0.999794 |
| Pc2_HyI | 24344 | 154.15 | 1.40 | 0.998679 |
| Pl3_Lht | 13931 | 37.06 | 1.11 | 0.999488 |
| Pd1_SjR | 21206 | 71.49 | 1.76 | 0.999699 |
| Pv2_HyI | 15956 | 71.29 | 1.82 | 0.9994 |
| Gr1_HyI | 15272 | 80.60 | 3.24 | 0.999859 |
| Hm2_SjR | 18278 | 43.35 | 0.85 | 0.999424 |
| Pv1_HyI | 14455 | 234.77 | 3.06 | 0.994043 |
| Hm1_SjR | 11732 | 68.42 | 1.09 | 0.998673 |
| Gf1_DyB | 26670 | 60.22 | 2.15 | 0.999805 |
| Pl1_SjR | 10675 | 30.79 | 2.15 | 0.99981 |
| Pl5_HyI | 28829 | 160.13 | 2.56 | 0.99936 |
| Pc1_HyI | 15994 | 149.62 | 2.82 | 0.996188 |
| Gr2_DyB | 27353 | 95.98 | 2.54 | 0.999612 |
| Gr5_XyR | 17762 | 146.04 | 2.09 | 0.998115 |
| Fp1_Lht | 11128 | 56.01 | 2.20 | 0.999227 |
| Pd2_SjR | 18503 | 126.37 | 1.73 | 0.999346 |
| Pl4_SjR | 27655 | 72.33 | 1.85 | 0.999812 |
| Gr4_DyB | 23211 | 100.09 | 2.29 | 0.999731 |
| Pc2_SjR | 23953 | 129.75 | 1.97 | 0.99868 |
| Pl4_Lht | 15498 | 40.17 | 1.63 | 0.999736 |
| Fp3_WzI | 23281 | 293.57 | 4.03 | 0.999658 |
| Me2_SjR | 22700 | 59.97 | 1.10 | 0.999672 |
| Ar2_XyR | 19973 | 129.71 | 1.70 | 0.998679 |
| Ar1_XyR | 15170 | 172.75 | 2.95 | 0.99721 |
| Pl3_HyI | 14029 | 42.29 | 2.42 | 0.999708 |
| Pl2_SjR | 14764 | 31.54 | 2.29 | 0.999931 |
| Ar3_XyR | 20847 | 286.85 | 2.80 | 0.995584 |
| Pl2_HyI | 16341 | 74.87 | 2.19 | 0.999112 |
| Gr3_DyB | 14776 | 33.30 | 1.10 | 0.999632 |
| Pl3_SjR | 12523 | 121.88 | 0.58 | 0.998599 |
| Gr2_HyI | 12389 | 153.21 | 3.67 | 0.998254 |
| Pl2_Lht | 11960 | 202.85 | 3.24 | 0.998922 |
| Pl1_Lht | 17089 | 85.62 | 1.96 | 0.999174 |
| Pe1_SjR | 20999 | 137.21 | 3.08 | 0.999586 |
| Fp2_WzI | 19846 | 201.63 | 4.12 | 0.999731 |
| Pv2_Lht | 13950 | 55.35 | 1.34 | 0.999556 |
| Pl1_HyI | 13821 | 123.22 | 4.08 | 0.999686 |
| Pl2_XyR | 21595 | 52.96 | 1.81 | 0.999806 |

^#^The letters before the underscore are the initials of the coral genus and species, which represent the species of corals including Pl (*Porites lutea*), Pv (*Plesiastrea versipora*), Fp (*Favia palauensis*), Gr (*Goniastrea retiformis*), Pe (*Pocillopora eydouxi*), Ar (*Acropora rosaria*), Me (*Montipora efflorescens*), Hm (*Hydnophora microconos*), Gf (*Galaxea fascicularis*), Pd (*Pavona decussata*), and Pc (*Plesiastrea curta*). The numbers indicate the order of coral individuals. The letters after the underscore represent the abbreviations of coral reef locations including DyB (Daya Bay), HyI (Huangyan Island), Lht (Luhuitou), SjR (Sanjiao Reef), XyR (Xinyi Reef), and WzI (Weizhou Island).

**Table S2** Number of diazotrophs associated with different coral colonies at different taxonomic levels.

| Coral code | Phylum | Class | Order | Family | Genus | Species | OTU |
| --- | --- | --- | --- | --- | --- | --- | --- |
| Gr3_HyI | 5 | 9 | 11 | 11 | 12 | 12 | 68 |
| Pv1_Lht | 4 | 8 | 10 | 10 | 10 | 11 | 64 |
| Pv3_HyI | 5 | 10 | 14 | 16 | 17 | 17 | 202 |
| Pl4_HyI | 6 | 11 | 16 | 18 | 19 | 19 | 179 |
| Pv2_WzI | 5 | 8 | 10 | 10 | 10 | 10 | 34 |
| Pc1_SjR | 5 | 10 | 15 | 16 | 16 | 16 | 146 |
| Gr2_XyR | 5 | 10 | 12 | 13 | 15 | 15 | 130 |
| Pv3_Lht | 3 | 4 | 5 | 5 | 5 | 5 | 21 |
| Gf2_DyB | 5 | 10 | 16 | 16 | 17 | 17 | 143 |
| Pv3_WzI | 3 | 7 | 9 | 9 | 9 | 9 | 103 |
| Pl1_XyR | 5 | 6 | 7 | 9 | 11 | 11 | 74 |
| Pd2_DyB | 5 | 8 | 10 | 11 | 12 | 12 | 88 |
| Pl4_WzI | 7 | 10 | 14 | 16 | 17 | 17 | 215 |
| Hm1_DyB | 4 | 7 | 8 | 8 | 10 | 10 | 50 |
| Gr4_XyR | 4 | 9 | 14 | 14 | 15 | 15 | 66 |
| Gr1_DyB | 6 | 9 | 13 | 13 | 14 | 14 | 52 |
| Pl3_XyR | 4 | 9 | 12 | 12 | 12 | 12 | 153 |
| Pc3_SjR | 5 | 8 | 9 | 10 | 11 | 11 | 132 |
| Fp1_SjR | 3 | 7 | 9 | 9 | 9 | 9 | 96 |
| Pl3_WzI | 4 | 7 | 8 | 8 | 8 | 9 | 88 |
| Me1_SjR | 4 | 9 | 11 | 12 | 12 | 12 | 101 |
| Gr1_XyR | 5 | 9 | 11 | 11 | 11 | 11 | 50 |
| Gr3_XyR | 6 | 11 | 18 | 19 | 21 | 21 | 280 |
| Pl2_WzI | 4 | 8 | 16 | 16 | 17 | 17 | 117 |
| Fp1_XyR | 5 | 9 | 11 | 13 | 13 | 13 | 112 |
| Fp1_WzI | 6 | 9 | 14 | 15 | 18 | 18 | 93 |
| Pl1_WzI | 7 | 11 | 22 | 25 | 28 | 30 | 205 |
| Pl4_XyR | 4 | 9 | 14 | 15 | 16 | 16 | 52 |
| Pv1_WzI | 5 | 9 | 12 | 12 | 12 | 12 | 75 |
| Pd1_DyB | 4 | 5 | 6 | 6 | 7 | 7 | 32 |
| Pc2_HyI | 3 | 4 | 7 | 7 | 8 | 8 | 61 |
| Pl3_Lht | 4 | 6 | 6 | 6 | 6 | 6 | 24 |
| Pd1_SjR | 5 | 8 | 11 | 11 | 12 | 12 | 67 |
| Pv2_HyI | 6 | 9 | 13 | 13 | 13 | 13 | 62 |
| Gr1_HyI | 4 | 6 | 9 | 11 | 11 | 11 | 80 |
| Hm2_SjR | 5 | 8 | 9 | 9 | 10 | 10 | 33 |
| Pv1_HyI | 5 | 9 | 12 | 13 | 13 | 13 | 179 |
| Hm1_SjR | 7 | 9 | 11 | 11 | 11 | 11 | 54 |
| Gf1_DyB | 5 | 6 | 7 | 7 | 8 | 8 | 55 |
| Pl1_SjR | 4 | 5 | 6 | 6 | 6 | 6 | 30 |
| Pl5_HyI | 7 | 12 | 19 | 20 | 21 | 21 | 144 |
| Pc1_HyI | 6 | 10 | 16 | 19 | 20 | 21 | 154 |
| Gr2_DyB | 5 | 10 | 11 | 11 | 12 | 12 | 86 |
| Gr5_XyR | 4 | 9 | 14 | 15 | 15 | 15 | 125 |
| Fp1_Lht | 5 | 8 | 9 | 9 | 10 | 10 | 49 |
| Pd2_SjR | 7 | 12 | 16 | 16 | 17 | 17 | 121 |
| Pl4_SjR | 6 | 10 | 14 | 14 | 15 | 15 | 69 |
| Gr4_DyB | 5 | 8 | 10 | 10 | 11 | 11 | 96 |
| Pc2_SjR | 3 | 7 | 11 | 12 | 12 | 12 | 104 |
| Pl4_Lht | 3 | 4 | 5 | 5 | 5 | 5 | 37 |
| Fp3_WzI | 5 | 9 | 16 | 19 | 21 | 23 | 291 |
| Me2_SjR | 4 | 8 | 11 | 12 | 12 | 12 | 57 |
| Ar2_XyR | 6 | 8 | 11 | 12 | 14 | 14 | 111 |
| Ar1_XyR | 6 | 11 | 17 | 18 | 18 | 18 | 136 |
| Pl3_HyI | 6 | 9 | 12 | 12 | 12 | 12 | 36 |
| Pl2_SjR | 4 | 6 | 7 | 7 | 7 | 7 | 31 |
| Ar3_XyR | 7 | 10 | 11 | 14 | 14 | 14 | 189 |
| Pl2_HyI | 4 | 7 | 10 | 11 | 11 | 11 | 62 |
| Gr3_DyB | 5 | 7 | 7 | 7 | 8 | 8 | 30 |
| Pl3_SjR | 3 | 6 | 7 | 7 | 7 | 7 | 34 |
| Gr2_HyI | 5 | 10 | 15 | 16 | 17 | 17 | 143 |
| Pl2_Lht | 5 | 10 | 15 | 16 | 17 | 18 | 199 |
| Pl1_Lht | 6 | 9 | 12 | 14 | 15 | 16 | 77 |
| Pe1_SjR | 7 | 11 | 16 | 16 | 16 | 16 | 133 |
| Fp2_WzI | 6 | 10 | 14 | 14 | 14 | 14 | 200 |
| Pv2_Lht | 6 | 9 | 10 | 11 | 12 | 13 | 52 |
| Pl1_HyI | 6 | 11 | 15 | 18 | 19 | 19 | 121 |
| Pl2_XyR | 5 | 8 | 10 | 10 | 10 | 10 | 51 |
